# Hydrothermal Synthesis of Water-Dispersible Fluorescent Carbon Dots from Renewable Plant Precursors: Antibacterial Activity and Comparative Characterization

**DOI:** 10.3390/nano16201328

**Published:** 2026-10-20

**Authors:** Nataliia Dudchenko, Arulappan Durairaj, Heba Shaban Agbaria, Elena Afrimzon, Ulugbek Saidvaliev, Dror Fixler, Sivan Shoshani, Ilana Perelshtein, Ehud Banin

**Affiliations:** 1 Bar-Ilan Institute of Nanotechnology & Advanced Materials (BINA), Bar Ilan University, Ramat Gan 5290002, Israel; chemdraj@gmail.com (A.D.); heba12heba12@gmail.com (H.S.A.); elena.afrimzon@biu.ac.il (E.A.); sivan.shoshani@biu.ac.il (S.S.); ilana.perelshtein@biu.ac.il (I.P.); ehud.banin@biu.ac.il (E.B.); 2 Faculty of Engineering, School of Biomedical Engineering, Tel Aviv University, Tel Aviv 6997801, Israel; ulugbeks@tauex.tau.ac.il (U.S.); fixeled@tauex.tau.ac.il (D.F.)

**Keywords:** carbon dots, renewable biomass, hydrothermal synthesis, fluorescent nanomaterials, antibacterial activity, sustainable nanotechnology

## Abstract

We present here a one-pot hydrothermal synthesis of fluorescent carbon dots (CDs) using olive, rosemary, sage, and thyme leaves as a carbon source. The hydrothermal reaction was conducted at 180 °C for 12 h, thereby converting biomass into water-dispersible CDs without a passivating agent. The average size of the CDs, determined by TEM, was 3–6 nm, with a spherical morphology. All synthesized CDs displayed excitation-dependent fluorescence, with emission maxima at 400–440 nm (blue) with excitation at 340–370 nm. FLIM measurements yielded similar fluorescence lifetimes of approximately 5 ns in aqueous solutions of CDs. FTIR spectra indicated surface hydroxyl and nitrogen-containing functionalities that may contribute to photoluminescence. The antibacterial activity of the as-synthesized CDs against a panel of Gram-positive and Gram-negative bacteria (*Staphylococcus aureus*, *Listeria innocua*, *Escherichia coli*, *Pseudomonas aeruginosa*, methicillin-resistant *S. aureus* (MRSA), multidrug-resistant (MDR) *E. coli*, and *Klebsiella pneumoniae* (MDR)) was investigated. It was shown that biomass-derived CDs exhibit antibacterial activity, being more effective against Gram-positive bacteria, with MIC values as low as 0.625 mg/mL for olive-, rosemary-, and thyme-derived CDs against *S. aureus* and MRSA. These findings suggest that the developed CDs are fluorescent materials offering viable possibilities in biomedical research, as well as antimicrobial agents.

## 1. Introduction

Carbon dots (CDs) are zero-dimensional, carbon-based nanomaterials typically smaller than 10 nm. They were first reported in 2004 by Xu et al. [1], who identified them as fluorescent by-products during the purification of single-walled carbon nanotubes derived from arc-discharge soot. Since then, CDs have gained significant attention because of their tunable photoluminescence, high water dispersibility, chemical stability, and reported biocompatibility, which support applications in biomedicine [2,3], biosensing [4,5,6,7,8], theranostics [9,10], antimicrobial systems [11], and food preservation/packaging [12,13].

Over the past twenty years, research into the synthesis, properties, and applications of CDs has grown exponentially. Generally, CDs can be produced through two main approaches: top-down and bottom-up strategies [14]. Top-down methods involve breaking down bulk carbon materials and include techniques such as arc discharge, laser ablation, electrochemical oxidation, chemical oxidation, and ultrasonic treatment [15]. Conversely, bottom-up approaches rely on converting molecular precursors into carbon structures and include methods like microwave-assisted synthesis, thermal decomposition, hydrothermal treatment, template techniques, and plasma synthesis [16]. Each approach offers specific advantages and limitations, and selecting a synthesis method is largely dependent on the intended application [17].

A wide variety of carbon precursors have been explored for CD production, including synthetic chemicals (such as polyethylene glycol [18], glucosamine [19], citric acid [20], and chitosan [21]), natural resources (like medicinal plants and marine polysaccharides [22,23]), and bio-waste streams [24]. Among these, biomass-derived precursors are especially attractive due to their renewability, low cost, and natural heteroatom content. Natural biomass contains diverse organic compounds such as proteins, amino acids, and carbohydrates, along with heteroatoms such as nitrogen and sulfur, which can facilitate in situ surface doping and functionalization, thereby modifying the optical and biological properties of CDs. Accordingly, hydrothermal carbonization of biomass is widely used as a bottom-up route to CDs, with precursor composition and resulting surface functionality influencing optical properties and biological interactions, including antimicrobial activity [25].

Mediterranean medicinal plants such as olive (Olea europaea) leaves, rosemary (Salvia rosmarinus), sage (Salvia officinalis), and thyme (Thymus vulgaris) are rich in polyphenols, flavonoids, terpenoids, and phenolic acids, and contain compounds with reported antibacterial and antioxidant activities. In their bulk forms—such as extracts or essential oils—these plants display antimicrobial activity largely attributed to specific bioactive molecules like oleuropein, rosmarinic acid, thymol, and carvacrol [26,27,28]. However, the activity and formulation of plant extracts or essential oils can be limited by solubility, volatility, stability, and compositional variability. In contrast, converting plant biomass into CDs via hydrothermal processes allows the transformation of complex phytochemicals into water-dispersible nanostructures bearing surface functional groups. Such nanoscale restructuring can alter interactions with bacterial cells through changes in surface area, colloidal behavior, and surface chemistry [29]. Despite the established antimicrobial properties of these plants in their bulk form [26,27,28], direct, controlled comparisons between the parent plant materials and the corresponding biomass-derived CDs remain limited. In this context, systematic comparative investigations of how various green precursors influence the properties of the resulting Carbon Dots, obtained by hydrothermal carbonization, and, consequently, their antibacterial activity, pose both scientific and practical questions. Such comparisons are particularly important in the fight against multidrug-resistant (MDR) pathogens, where new antimicrobial approaches are urgently needed.

This study reports the one-pot hydrothermal synthesis of fluorescent, water-dispersible CDs from olive, rosemary, sage, and thyme leaves. We evaluated their antibacterial activity against a broad spectrum of Gram-positive and Gram-negative bacteria, including MDR strains. Overall, these results describe the physicochemical and antibacterial properties of plant-derived carbon nanomaterials and assess their potential as sustainable antimicrobial agents.

## 2. Materials and Methods

### 2.1. Materials

Olive leaves (*O. europaea*), rosemary (*S. rosmarinus*), sage (*S. officinalis*), and thyme (*T. vulgaris*) were used as plant materials in this study. Olive leaves and rosemary were collected from the campus area of Bar-Ilan University (Ramat Gan, Israel) during the spring–summer period, while sage and thyme were purchased from the local supermarket. The fresh leaves were stored in closed jars in a dry chemical cabinet before use. The botanical identities were assigned based on the plant species and the available source information. Ultrapure water was produced by the Milli-Q Direct Water Purification System. Strains of *Staphylococcus aureus* (ATCC 29213), *Listeria innocua* (ATCC 33090), *Escherichia coli* (ATCC 25922), *Pseudomonas aeruginosa* (PAO1 (B. Iglewski Strain) [30]), methicillin-resistant *S. aureus* (ATCC 43300), multidrug-resistant (MDR) *E. coli* (ATCC BAA-2452), and *Klebsiella pneumoniae* (MDR) (ATCC BAA-1898) were used.

### 2.2. Sample Preparation

CDs were produced by the one-pot hydrothermal method. The biomass was first washed with distilled water and dried at 60 °C for 48 h. Then, 6 g of dried biomass was dispersed in 60 mL of distilled water (the reactor capacity was 100 mL). For larger-scale production, 30 g of biomass was mixed with 300 mL of distilled water (the reactor capacity was 1000 mL). These mixtures were transferred into a Teflon-lined hydrothermal reactor and heated at 180 °C for 12 h. After cooling to room temperature, the reaction mixture was filtered through Whatman filter paper to remove large particles. The filtrate (50 mL) was then centrifuged at 9000 rpm (9056× *g*), cooled to 17 °C, for 20 min to remove insoluble residues. The supernatant was passed through a 0.22 µm syringe filter to eliminate any remaining particulates. The purified solution was washed with dichloromethane (1:1, *v*/*v*) and then freeze-dried for 72 h. The resulting dried carbon dots were stored at 4 °C until further use.

### 2.3. Sample Characterization

Fluorescence spectra were acquired on a Cary Eclipse fluorescence spectrophotometer (Varian, Palo Alto, CA, USA). High-resolution transmission electron microscopy (HR-TEM) samples were prepared by placing a drop of the aqueous CD solution onto a copper-coated grid and drying it overnight at room temperature. The HR-TEM images were acquired on JEOL JEM-2100 and JEM-F200 FEG transmission electron microscopes (JEOL Ltd., Peabody, MA, USA). Fourier transform infrared (FT-IR) spectra were recorded with a Nicolet iS10 spectrometer (Thermo Fisher Scientific, Madison, WI, USA) in the range of 500–4000 cm^−1^. Fluorescence lifetime measurements of CD solutions in distilled water were recorded with a frequency-domain Lambert Instruments FLIM LIFA system (Lambert Instruments, Groningen, The Netherlands) attached to an Olympus IX-81 inverted microscope with a 10× (NA = 0.4) objective (Olympus, Tokyo, Japan) at a temperature of 21 °C. The excitation source was a 468 nm modulated multi-light-emitting diode (LED) system controlled by a signal generator (Prior OptiScan II, Rockland, MA, USA). Multiple scanning frequencies were utilized for sample excitation within the range of 30–60 MHz; 5 consecutive measurements of independent batches were taken for each sample. Fluorescence emission was detected through the FITC detection channel of the LIFA FLIM system, which detects emission. Fluorescence-lifetime data were analyzed by LIFA FLIM v1.2.26 with a single-exponential fitting model (fluorescence lifetime measurement range of the setup is 0.1–100 ns). UV-VIS spectra were recorded on a Cary 60 UV-VIS spectrophotometer (Agilent Technologies, Santa Clara, CA, USA). X-ray Photoelectron Spectroscopy (XPS) spectra were acquired on a Thermo Scientific Nexsa spectrometer (Thermo Fisher Scientific, Waltham, MA, USA). Powder X-ray diffraction (XRD) measurements were performed using a SmartLab X-ray diffractometer (Rigaku Corporation, Tokyo, Japan) equipped with a high-flux 9 kW Cu X-ray source (Cu Kα radiation, λ = 1.5406 Å). The diffraction patterns were recorded over a 2θ range of 10–70°.

### 2.4. Antibacterial Activity

The antibacterial activity of the samples was assessed by measuring the minimum inhibitory concentration (MIC) and minimum bactericidal concentration (MBC). MIC values were obtained using the broth microdilution method. Briefly, bacterial strains were grown overnight at 37 °C in Difco™ LB broth (LB) or Difco™ Brain Heart Infusion (BH) broth (for *L. innocua*). Aliquots of 200 µL of the tested samples at twofold serially diluted concentrations were mixed with 200 µL of bacterial inoculum in appropriate medium (final bacterial concentration was approximately 1 × 10^5^ cell/mL) in a 96-well polypropylene microplate. The plates were incubated at 37 °C with shaking at 110 rpm overnight. Sterility-control wells containing medium without bacteria were kept under the same conditions. The positive growth control and each carbon dot concentration without bacterial inoculation were also tested. Bacterial growth was measured by reading the optical density at 600 nm (OD_600_) with a microplate reader (Synergy, BioTek Instruments, Winooski, VT, USA). For each experimental well, the OD_600_ value measured for the corresponding carbon dot concentration without bacterial inoculation was subtracted to account for background absorbance resulting from the color and turbidity of the carbon dots and medium. The corrected OD_600_ values were then used to support the final determination of the MIC. OD_600_ values lower than 0.2 were considered no growth, and the MIC was determined to be the lowest concentration of the tested sample that showed no growth. The choice of medium was in accordance with the protocol established in our laboratory, which was determined to be optimal for the bacteria tested. To determine the MBC, samples were collected from the wells at the MIC and higher concentrations. A serial dilution was prepared, and the bacterial samples were plated by the drop plate method (detection limit < 10^2^ CFU/mL) onto LB agar. The plates were then incubated for 18 h at 37 °C, after which viable colonies were counted. The MBC was defined as the lowest concentration that resulted in a 99.9% reduction in bacterial concentration; as the initial bacterial concentration was 1 × 10^5^ cell/mL, samples containing 10^2^ CFU/mL or fewer were considered to represent ≥99.9% reduction in the viable bacterial population.

## 3. Results

### 3.1. Size, Morphology and Structure

**Figure 1:**
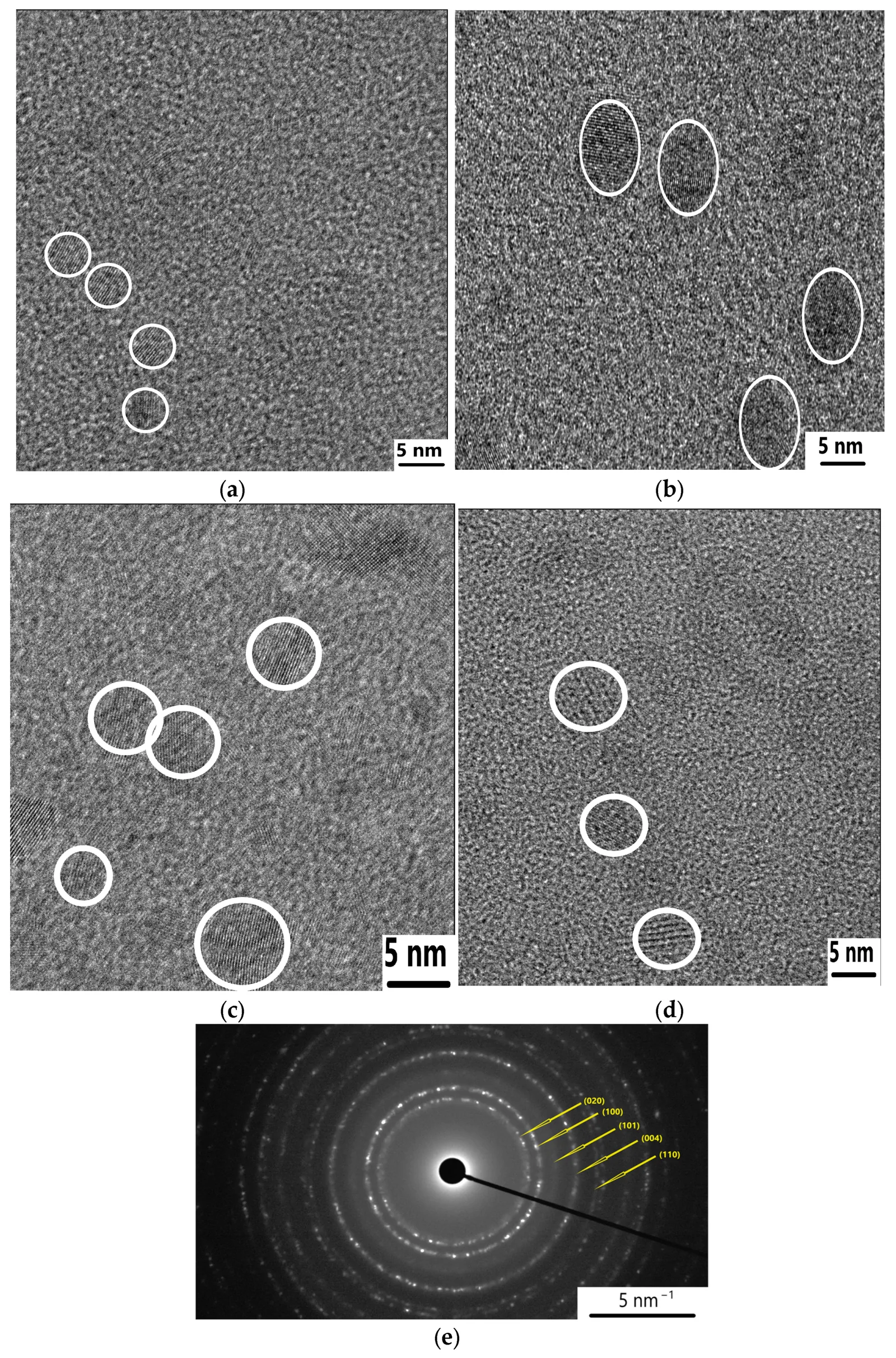
HR-TEM (JEOL Ltd., Peabody, MA, USA) images of CDs derived from: (**a**) Olive leaves; (**b**) Rosemary; (**c**) Sage; (**d**) Thyme. (**e**) A representative SAED image of CDs derived from Olive leaves.

The HR-TEM images of CDs derived from olive, rosemary, sage, and thyme are shown in Figure 1 (and in Supplementary Materials at other magnifications (Figures S1–S4)). The size distribution diagrams can be found in the Supplementary Materials (Figures S5–S8). These images indicate that all synthesized CDs are predominantly spherical and exhibit relatively uniform morphology.

The average particle sizes of the samples were analyzed using ImageJ 1.54g software (Table 1).

The obtained CDs exhibited nanoscale dimensions, with mean diameters in the range of 3–6 nm. Rosemary-derived CDs have the largest mean diameter, whereas thyme- and olive-derived CDs exhibit smaller sizes. The relatively narrow size distributions, as evidenced by the standard deviation values, suggest modest within-sample size dispersion after hydrothermal synthesis. Partial structural ordering of all synthesized CDs was observed in the selected area electron diffraction (SAED) images, with the measured d-spacings being consistent with the presence of graphitic-like domains (the representative SAED pattern for olive-derived CDs is shown in Figure 1e). The measured d-spacings obtained from Figure 1e (2.80, 2.41, 2.06, 1.71, and 1.46 Å) are characteristic of a disordered, graphitic-like carbon structure typically observed in carbon dots. In particular, the 2.80 lattice spacing is compatible with locally ordered graphitic domains within the disordered carbon structure. The 2.41 and 2.06 spacings are consistent with the graphitic carbon in-plane lattice spacing. The observed slight deviations of some lattice spacings from graphite may arise due to heteroatom incorporation and local defects. The presence of additional reflections below 1.8 Å further indicates the short-range ordered sp^2^ carbon regions. Overall, the diffuse and multiple-ring SAED pattern supports the assignment of partially graphitized, defective carbon dots rather than a well-defined crystalline phase such as graphite.

**Figure 2:**
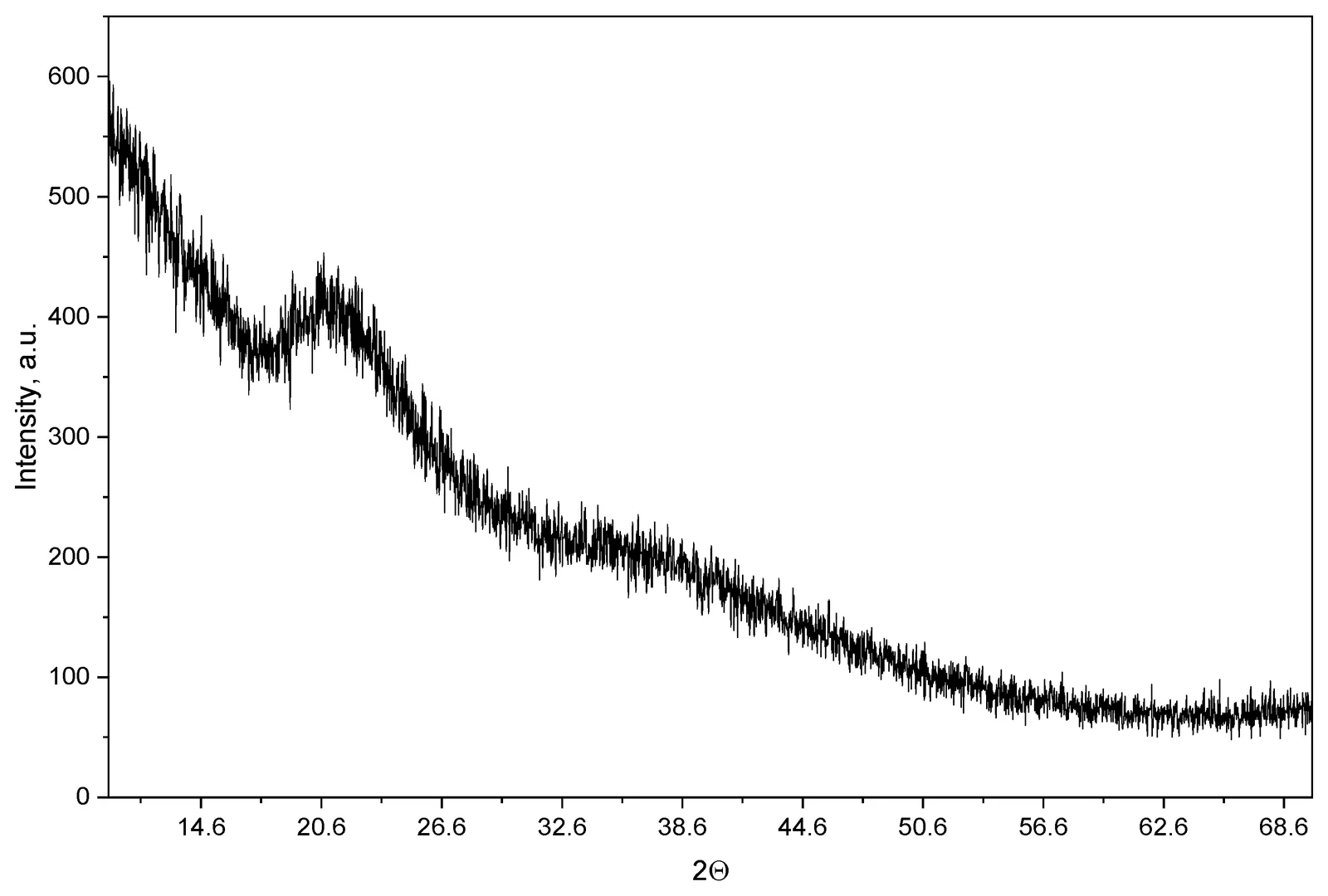
Powder XRD pattern of CDs derived from Olive leaves.

Powder XRD analysis was performed to further characterize the structural organization of the synthesized CDs. All four CD samples exhibited similar XRD profiles; therefore, the XRD pattern of olive-derived CDs is presented in Figure 2 as a representative example.

The XRD pattern exhibits a broad diffuse feature centered at approximately 2θ = 20–22°, without sharp diffraction peaks characteristic of a highly crystalline graphitic phase. This indicates predominantly disordered/amorphous carbon with a low degree of long-range structural order. At the same time, the SAED results indicate limited local ordering within the carbonaceous nanodomains. Therefore, the structure of synthesised CDs can be described as predominantly disordered/amorphous carbon containing limited locally ordered graphitic-like domains.

### 3.2. Fluorescent Properties

**Figure 3:**
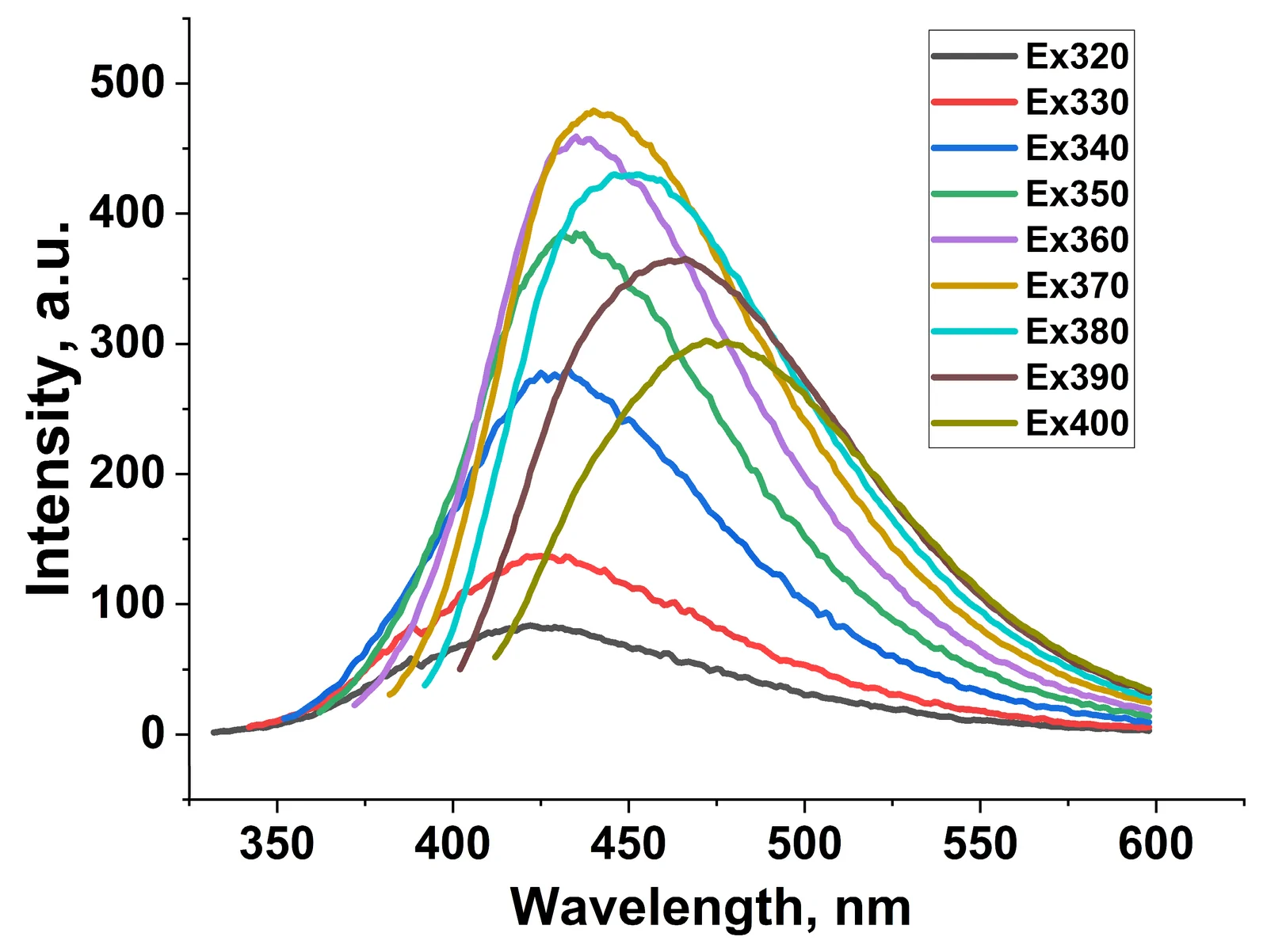
Fluorescence spectra of CDs derived from olive leaves at different excitation wavelengths.

The fluorescence emission of CDs is inherently complex and not yet fully understood, primarily due to multiple emissive pathways. These include intrinsic bandgap transitions of the carbon core, surface defect states, and molecular-like fluorophores formed during synthesis. Additionally, excitation-dependent behavior is often linked to heterogeneous particle-size distributions, diverse surface functional groups, and various energy-trap states, all of which lead to multiple radiative recombination channels. The relative importance of these mechanisms can vary with precursor composition and synthesis conditions, making the emission behavior more difficult to interpret. The synthesized CDs showed excitation-dependent blue fluorescence, with emission maxima ranging from 400 to 440 nm when excited at 340 to 370 nm, depending on the precursor used. Representative fluorescence spectra of olive-derived CDs are shown in Figure 3, and other spectra are shown in the Supplementary Materials (Figures S9–S11). The aqueous solution of the synthesized CDs appeared light yellow under daylight and exhibited bright blue fluorescence under UV light, indicating strong emission within the visible spectrum.

The excitation-dependent fluorescence behavior suggests the presence of multiple emissive states in the CDs. This phenomenon is often caused by a combination of intrinsic π–π* transitions in the carbon core and n–π* transitions involving surface functional groups such as hydroxyl, carbonyl, and carboxyl groups. Additionally, variations in particle size and surface chemistry likely create a range of energy levels and trap states, leading to wavelength-dependent emission. Surface defects and molecular fluorophores formed during hydrothermal carbonization also contribute to the overall photoluminescence. These factors collectively produce multiple radiative recombination pathways, making the emission mechanism complex and responsible for excitation-dependent behavior. The emission remained within the blue spectral region, while its excitation dependence is consistent with heterogeneous emissive states. Variations in precursor materials, especially natural polyphenols and organic compounds in biomass precursors, can further affect optical properties by introducing emissive centers or modifying surface functionality. These findings are consistent with previous studies on biomass-derived CDs, in which the interaction between the core structure and surface groups governs fluorescence properties [31,32]. The QY values of all synthesized CDs were found to be around 10–11% as estimated using quinine sulfate as a reference (calculation and UV-VI spectra given in the Supplementary Materials (Figures S12–S15)). Fluorescence lifetime (FLT) measurements provided results around 5 ns with a standard deviation of 0.2 ns for all four CD samples dissolved in deionized water (Table 2). Fluorescence intensity (FI) measurements were presented as a supportive measurement of CD emission, but not as a basis for quantitative comparison among preparations.

### 3.3. Surface Functionalization

**Figure 4:**
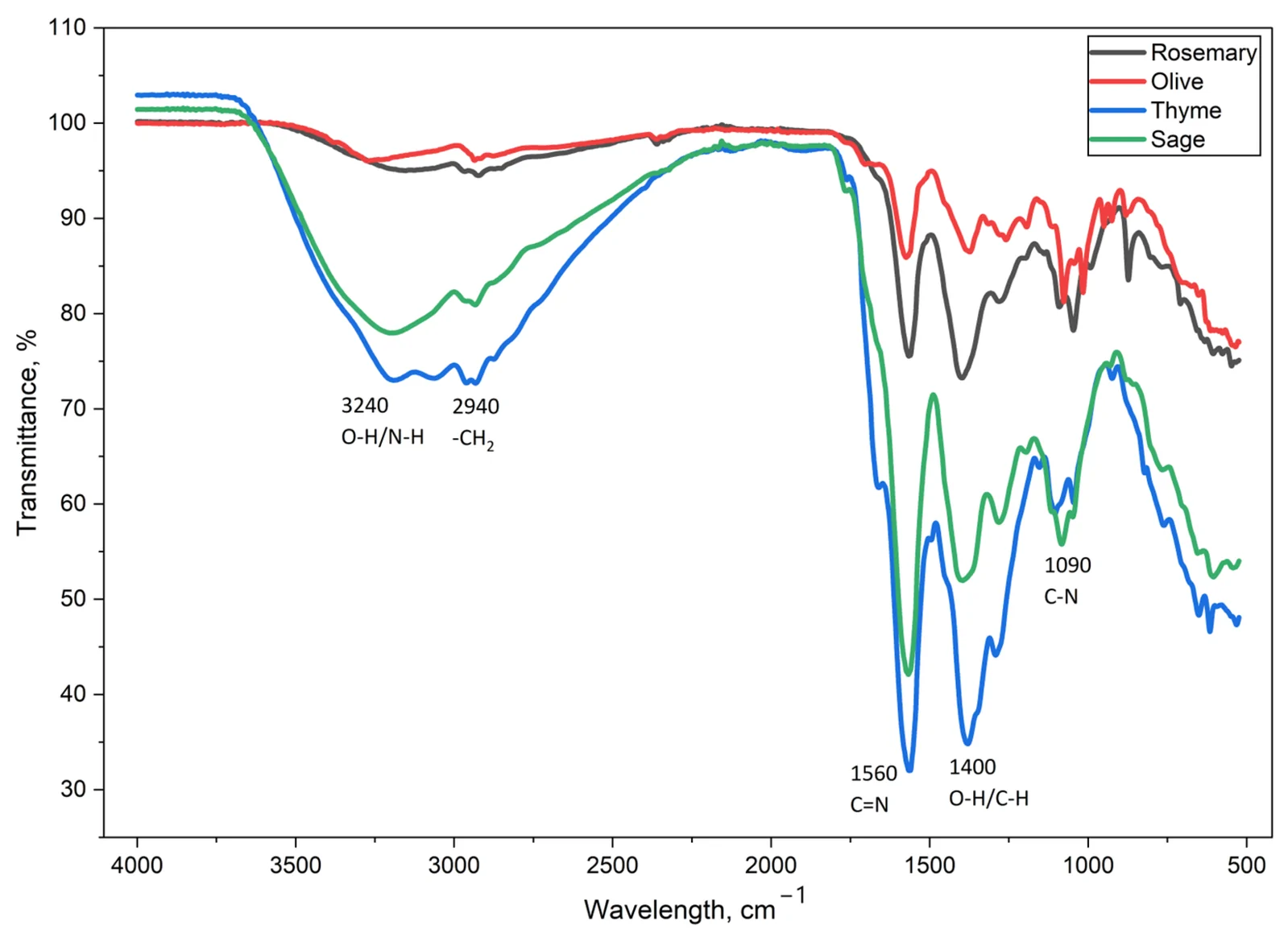
FT-IR spectra of CDs derived from Olive, Thyme, Sage, and Rosemary leaves.

FT-IR/XPS spectroscopy is commonly used to identify the functional groups present on the surface of CDs. As shown in Figure 4, all synthesized CDs display similar spectral features, indicating that they have comparable surface functionalities, regardless of the precursor used. A broad absorption band in the range of 3000–3500 cm^−1^ is attributed to the stretching vibrations of O–H and N–H bonds, consistent with the presence of hydroxyl and amine groups. The peak at 2940 cm^−1^ corresponds to the C–H stretching vibrations of aliphatic groups. Additionally, bands around 1560 cm^−1^ and 1100 cm^−1^ are associated with C=N and C–N stretching vibrations, respectively, which are consistent with nitrogen-containing surface functionalities. Peaks near 1400 cm^−1^ can be attributed to O–H bending and/or C–H vibrations.

These findings are in agreement with previous reports. For example, Hasan et al. [33] observed similar FT-IR features in biomass-derived N-doped CDs, including broad O–H/N–H bands and characteristic C=N and C–N vibrations, confirming the formation of nitrogen-functionalized carbon structures.

**Figure 5:**
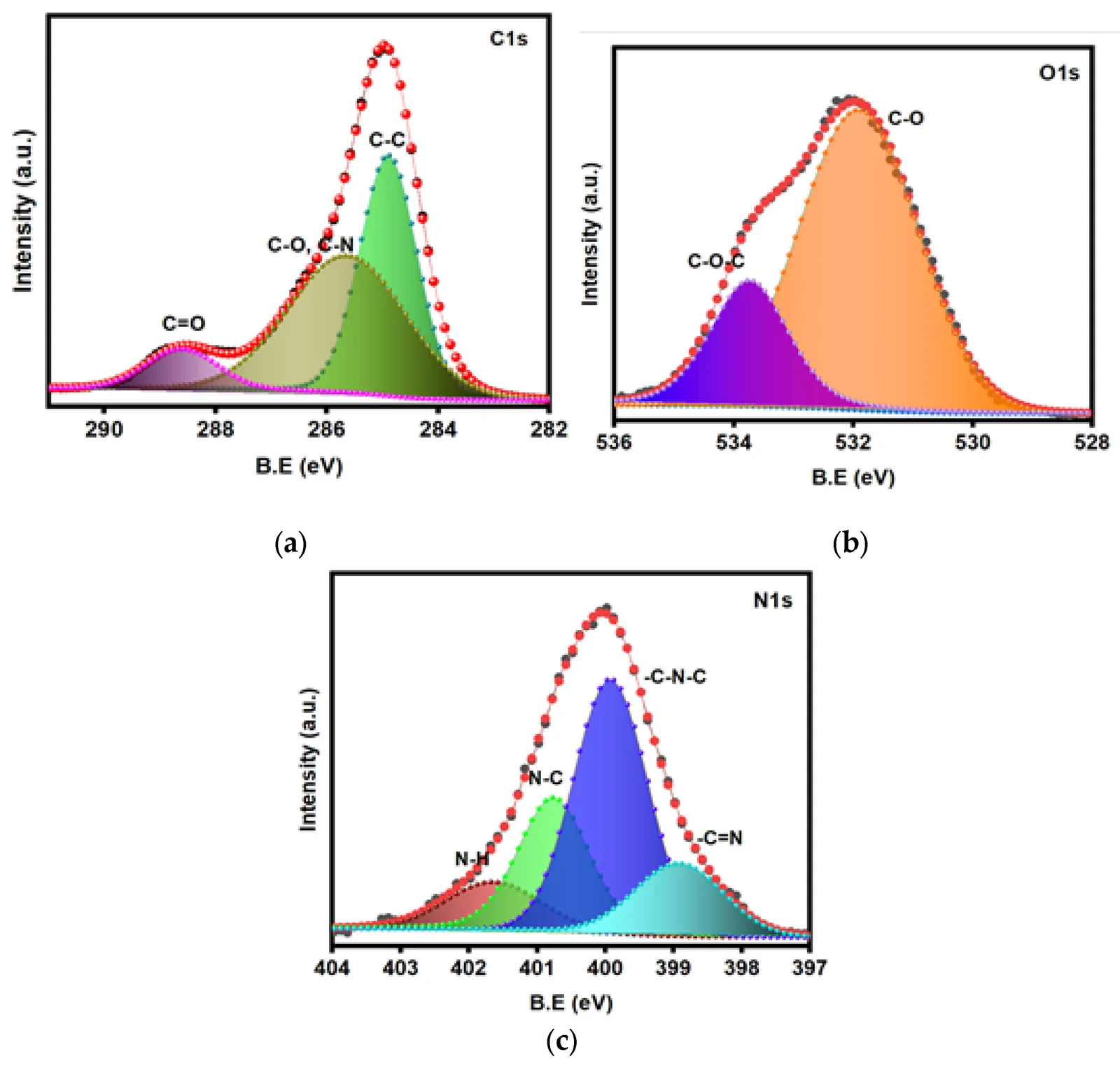
XPS spectra of Olive-derived CDs with peak deconvolution: (**a**) carbon (C1s), (**b**) oxygen (O1s), and (**c**) nitrogen (N1s) core-level spectra.

Representative XPS spectra of olive-derived CDs are displayed in Figure 5, and other spectra are shown in the Supplementary Materials (Figures S16–S18). The elemental analysis results of the CDs were made up of carbon (76.81%), oxygen (14.79%), and nitrogen (7.69%). In detail, the C 1s spectrum (Figure 5a) can be divided into three peaks at 284.9, 285.9, and 288.4 eV, which are ascribed to C=C, C–N, and C=O bonds, respectively. As shown in the high-resolution O 1s spectra (Figure 5b), the oxygen existed in two different states: C–O, and C–O–C (531.8 and 533.7 eV). In addition, the N 1s spectrum of the CDs (Figure 5c) could be decomposed into four peaks, such as -C=N (398.9 eV), C-N-C (400.1 eV), N-C (401 eV), and N-H (401.9 eV). These results established that the obtained CDs were doped with nitrogen and oxygen elements.

Bulk CHNS elemental analysis of the samples confirmed a substantial carbon content and the presence of nitrogen (see Supplementary Materials, Table S1).

### 3.4. Antibacterial Properties

The surface chemical composition and structural characteristics of the synthesized CDs are expected to play a role in their antibacterial activity [34,35]. The antibacterial performance of the CDs was further evaluated by MIC and MBC assays. All samples were tested against *S. aureus*, *L. innocua*, *E. coli*, *P. aeruginosa*, MRSA, *E. coli* MDR, and *K. pneumoniae* MDR (Table 3 and Table 4).

The antibacterial evaluation revealed that *S. aureus* and MRSA were the most sensitive bacteria, consistently showing the lowest MIC and MBC values across all CDs (MIC: 0.625–1.25 mg/mL; MBC: 1.25–2.5 mg/mL), indicating greater susceptibility than the Gram-negative strains tested. *L. innocua*, *E. coli*, and *P. aeruginosa* generally required higher concentrations for inhibition (MIC: 1.25–10 mg/mL; MBC: 2.5–10 mg/mL). The multidrug-resistant strains *E. coli* MDR and *K. pneumoniae* MDR required MIC and MBC values of 10–20 mg/mL, although these values overlapped with those observed for some non-MDR Gram-negative strains. The antibacterial activity showed some precursor-dependent differences: olive, rosemary, and thyme CDs showed similarly low MICs against Gram-positive bacteria (*S. aureus* and MRSA), whereas thyme CDs exhibited a higher MIC, but not MBC, for *P. aeruginosa*, and sage CDs showed moderate activity, with *L. innocua* not tested. The results are based on at least two biological replicates with two internal repeats.

The observed differences in antibacterial activity may partly reflect the structural and physicochemical properties of the synthesized CDs discussed above. The higher susceptibility of Gram-positive bacteria, particularly *S. aureus* and MRSA, may relate in part to their lack of an outer membrane, which may increase accessibility of the cell envelope to the CDs [35,36]. In contrast, Gram-negative bacteria such as *E. coli* and *P. aeruginosa* possess an additional outer membrane that acts as a permeability barrier, reducing the penetration and effectiveness of nanomaterials [36].

As reported by Giacometti et al. [37], the olive leaf extracts obtained by ultrasound-assisted extraction and conventional extraction have an inhibitory effect on the growth of *E. coli*, *Listeria* monocytogenes, *Yersinia enterocolitica*, and *S. aureus*, with MIC of 1.4–8.0 mg/mL and MBC of 4.0–12.0 mg/mL. They concluded that among the tested bacteria, olive leaf extracts demonstrated the highest antibacterial activity against *Y. enterocolitica* and *S. aureus* in extracts prepared by both methods. Antunes et al. [38] reported a minimum inhibitory concentration of 24 mg/mL for *E. coli* and 2.4 mg/mL for *S. aureus* for the olive leaf extract of the Frantoio cultivar. Abdelhamed et al. [39] investigated the antimicrobial activities of thyme essential oil (EO) using the broth microdilution method. They reported that the MIC and MBC of thyme EO against *Cutibacterium acnes* and *Staphylococcus epidermidis* were 26 mg/mL and 53 mg/mL, respectively. All four of our plant-derived CDs demonstrated measurable antibacterial activity. These results further demonstrate the antibacterial activity of the hydrothermally derived materials, particularly against Gram-positive bacteria.

To the best of our knowledge, only one study has addressed the synthesis and antibacterial applications of rosemary-derived carbon dots (CDs). Eskalen et al. [40] reported the synthesis of water-soluble, highly fluorescent CDs; however, these materials exhibited no antibacterial activity against *E. coli*, *Salmonella typhimurium*, *S. aureus*, *Bacillus subtilis*, or *Candida albicans*. In the case of olive-derived CDs, Nocito et al. [41] reported the preparation of CDs from olive solid waste collected in two Mediterranean regions, Puglia (CDs_P) and Calabria (CDs_C). Their antibacterial activity was assessed against *S. aureus* and *P. aeruginosa*. Notably, only CDs_C demonstrated antibacterial activity against *S. aureus*, with a minimum inhibitory concentration (MIC) of 0.36 mg/mL, whereas CDs_P showed no inhibitory effect against either bacterial strain.

## 4. Discussion

The findings of this work demonstrate that precursor-dependent variations in biomass composition may contribute to differences in nucleation, growth, surface chemistry, and the optical and antibacterial properties of the synthesized CDs. The observed differences in particle size among the samples may reflect differences in the chemical composition of the biomass precursors. Consistent with literature reports, differences in the content of polyphenols, carbohydrates, and other organic constituents may influence the nucleation and growth processes during carbonization, leading to CDs with distinct sizes and surface structures [28]. The smaller particle sizes observed for olive- and thyme-derived CDs may reflect precursor-dependent differences in carbonization and nucleation, although these mechanisms were not directly measured.

The nanoscale dimensions (<10 nm) and quasi-spherical morphology observed across all samples are consistent with the typical structural features of CDs synthesized via hydrothermal routes [42]. Importantly, the particle size is known to influence the optical properties of CDs, including fluorescence behaviour, through contributions from core structure and surface states [43]. In this context, the smaller CDs may exhibit more pronounced surface-state-related emission, while larger particles may show increased contribution from core-related electronic transitions. In addition, the nanoscale size of the CDs provides a high surface-to-volume ratio. Smaller particles, in particular, may exhibit enhanced antibacterial effects due to their increased surface reactivity and ability to penetrate or absorb onto bacterial membranes, contributing to membrane disruption and leakage of intracellular components [44].

A qualitative association between structural characteristics and optical behavior is further supported by the HR-TEM and fluorescence spectroscopy results. As noted, all samples exhibited excitation-dependent blue emission in the range of 400–440 nm, characteristic of carbon dots with heterogeneous emissive sites. Additionally, the variation in particle size distribution, indicated by the standard deviation values, suggests structural heterogeneity. This aligns with the observed broad and excitation-dependent emission profiles. Such heterogeneity results in a distribution of emissive energy levels, allowing for different radiative recombination pathways depending on the excitation wavelengths used [45]. The similar FLT values, clustered around 5.0 ns for all four CD types, indicate that the excited-state relaxation dynamics are largely conserved across the different biomass precursors. This suggests that the hydrothermal process generates similar dominant emissive centers, despite modest differences in particle size. The slightly higher mean FLT observed for rosemary- and sage-derived CDs should be interpreted cautiously, since the differences are small relative to the reported standard deviation. It is also important to consider that, in addition to size effects, differences in precursor composition (olive leaves, rosemary, sage, and thyme) may contribute to variations in surface chemistry, including the types and abundances of functional groups. These surface functionalities can influence the photoluminescence properties by introducing surface states and molecular-like fluorophores formed during hydrothermal treatment. Furthermore, the excitation-dependent fluorescence behaviour indicates the presence of multiple surface defect states and active sites, which could be the possible contributing factors of the generation of reactive oxygen species (ROS) under light irradiation [46]. According to the literature data, the formation of ROS, such as singlet oxygen or hydroxyl radicals, can induce oxidative stress in bacterial cells, leading to damage of cellular components, including lipids, proteins, and DNA [47].

The presence of oxygen- and nitrogen-containing surface functionalities, as revealed by FT-IR and XPS analysis, is consistent with the excitation-dependent fluorescence behavior discussed above. These surface groups are known to introduce defect states and energy traps, which play a key role in the photoluminescence of CDs by enabling multiple radiative recombination pathways [48]. This is consistent with the excitation-dependent blue emission observed in the 400–440 nm range. The combination of nanoscale dimensions (3–6 nm) and rich surface chemistry enhances the surface-to-volume ratio, thereby amplifying the contribution of surface states to the overall emission.

In addition to their optical properties, the synthesized CDs exhibit measurable antibacterial activity against the tested bacterial panel. The materials demonstrate activity against both Gram-positive and Gram-negative bacteria, including clinically relevant resistant strains. Although a conventional antimicrobial positive control was not included in this study, the MIC and MBC assays allowed a quantitative comparison of the antibacterial activity of the four CD preparations under identical experimental conditions. Collectively, these results establish a comparative activity profile and activity against resistant pathogens, supporting further evaluation of biomass-derived carbon dots for antimicrobial applications.

This behavior may be related to their oxygen- and nitrogen-containing surface functionalities that are possible contributing factors which can affect surface charge, hydrophilicity, and interaction with bacterial membranes. In particular, biomass rich in polyphenols and bioactive compounds is likely to yield CDs with more reactive surface sites, thereby promoting stronger interactions with microbial cells and enhancing antibacterial efficacy [49].

Despite the reduced susceptibility of multidrug-resistant strains, the ability of CDs to inhibit their growth at higher concentrations is notable, highlighting the potential of these nanomaterials as alternative antimicrobial agents. Unlike conventional antibiotics, the possible factors contributing to the antibacterial activity of CDs are thought to occur through multiple mechanisms, including physical disruption of cell membranes, chemical interactions mediated by surface functional groups, and the generation of oxidative stress [50,51]. Such multimodal antibacterial mechanisms have been proposed for some CDs in the literature. Due to these properties, CDs are increasingly integrated into polymers, textiles, medical-device coatings, and food-packaging materials to produce antibacterial coatings with the purpose of improving hygienic performance and reducing microbial contamination [52,53]. Moreover, the combination of optical and antibacterial functionalities enables the development of multifunctional smart coatings capable of simultaneous microbial inhibition and fluorescence-based surface monitoring [34]. CDs, demonstrating potential as antioxidant agents, UV-protective additives, and nanocarriers for active ingredients, could find potential future applications in cosmetics [54]. Their ability to scavenge reactive oxygen species may enhance anti-aging and skin-protective formulations [55,56]. Additionally, fluorescent CDs can be used in innovative cosmetic systems to provide a color-changing effect in response to external factors (UV light, skin pH, environmental conditions, etc.) [57]. Carbon dots derived from natural or biomass-based precursors are especially appealing for creating environmentally friendly and sustainable cosmetic formulations.

Collectively, this work demonstrated a combination of excitation-dependent fluorescence, nanoscale dimensions, and antibacterial activity across the tested panel that supports further investigation of these biomass-derived nanomaterials for antimicrobial applications.

## 5. Conclusions

In this study, carbon dots were successfully synthesized via a one-pot hydrothermal method using biomass precursors, including olive leaves, rosemary, sage, and thyme. The obtained CDs exhibited spherical morphology with nanoscale dimensions (3–6 nm), as confirmed by HR-TEM analysis, and showed similar surface functional groups that include oxygen- and nitrogen-containing moieties, as revealed by FT-IR spectroscopy.

All synthesized CDs exhibited excitation-dependent blue fluorescence, consistent with contributions from the carbon core structure and surface states. Fluorescence lifetime measurements revealed similar decay characteristics for all four CD samples, with mean lifetimes in the range of 4.9–5.1 ns and a standard deviation of approximately 0.2 ns. The comparable lifetime values suggest that, despite differences in biomass precursor and particle size, the dominant emissive states formed during hydrothermal carbonization are broadly similar among the samples. In contrast, fluorescence intensity showed moderate precursor-dependent variation, which may reflect differences in concentration, surface chemistry, or emissive-site density. These findings indicate that FLIM provides a complementary photophysical measurement of the synthesized CDs and complements the excitation-dependent fluorescence spectra. The optical data are consistent with contributions from both particle size and precursor-dependent surface chemistry, highlighting the importance of biomass composition in tailoring the functionalities of these sustainable nanomaterials.

The antibacterial assays demonstrated measurable inhibitory and bactericidal activity of the CD preparations under the tested conditions, with greater activity against Gram-positive bacteria, particularly *S. aureus* and MRSA, and substantially lower activity against several Gram-negative and multidrug-resistant strains.

This work demonstrates that biomass-derived CDs combine excitation-dependent fluorescence with antibacterial activity, and could find potential future applications in antimicrobial coatings and food packaging. Furthermore, the use of natural precursors provides a renewable-precursor route for the development of functional nanomaterials.

## Figures and Tables

**Table 1:** The average particle sizes of the synthesized CDs.

Size, nm	Olive Leaves	Rosemary	Sage	Thyme
Mean	3.25	5.75	4.7	3.0
SD	0.8	1.27	1.3	0.7

**Table 2:** Fluorescence lifetime measurement results.

FL Parameters	Olive Leaves	Rosemary	Sage	Thyme
FLT mean, ns	4.91	5.01	5.1	4.9
STD, ns	0.2	0.2	0.2	0.2
FI mean, a.u.	25,168	24,132	26,351	25,423
STD, a.u.	1345	1256	1476	1328

Note: all results were obtained with χ^2^ (chi-square) estimates of less than 15.

**Table 3:** MIC (mg/mL) results summary of Carbon Dots derived from Olive, Sage, Rosemary, and Thyme leaves.

Source	Bacteria						
	*S. aureus*	*L. innocua*	MRSA	*P. aeruginosa*	*E. coli*	*E. coli* MDR	*K. pneumoniae* MDR
Olive	0.625–1.25	1.25–2.5	0.625–1.25	5	5–10	10–20	10–20
Sage	2.5	–	0.625–1.25	5	10	10–20	20
Rosemary	0.625–1.25	1.25–2.5	0.625	5	10	20	10–20
Thyme	0.625	–	0.625	5–10	10	10	10–20

Note: “–” indicates the bacteria were not tested.

**Table 4:** MBC (mg/mL) results summary of Carbon Dots derived from Olive, Sage, Rosemary, and Thyme leaves.

Source	Bacteria						
	*S. aureus*	*L. innocua*	MRSA	*P. aeruginosa*	*E. coli*	*E. coli* MDR	*K. pneumoniae* MDR
Olive	1.25–2.5	2.5	1.25–2.5	10	5–10	20	20
Sage	2.5	–	2.5	10	10	20	20
Rosemary	1.25	2.5–5	0.625	10	10	20	20
Thyme	1.25	–	0.625–1.25	10	10	10–20	20

Note: “–” indicates the bacteria were not tested.

## Data Availability

The original contributions presented in this study are included in the article and Supplementary Material. Further inquiries can be directed to the corresponding author.

## Supplementary Materials

The following supporting information can be downloaded at: https://www.mdpi.com/article/10.3390/nano16201328/s1, Figure S1: TEM images of Olive-derived CDs at various magnifications: (a) scale bar = 100 nm, (b) scale bar = 10 nm; Figure S2: TEM images of Rosemary-derived CDs at various magnifications: (a) scale bar = 50 nm, (b) scale bar = 10 nm; Figure S3: TEM images of Thyme-derived CDs at various magnifications: (a) scale bar = 20 nm, (b) scale bar = 2 nm; Figure S4: TEM images of Sage-derived CDs at various magnifications: (a) scale bar = 50 nm, (b) scale bar = 5 nm; Figure S5: Size distribution diagram of Olive-derived CDs. The number of particles is ~60, the number of micrographs is 15, and the number of independent synthesis batches analyzed is 6. Histograms were generated from measurements of particles selected from multiple TEM images; Figure S6: Size distribution diagram of Rosemary-derived CDs. The number of particles is ~90, the number of micrographs is 25, and the number of independent synthesis batches analyzed is 5. Histograms were generated from measurements of particles selected from multiple TEM images; Figure S7: Size distribution diagram of Thyme-derived CDs. The number of particles is ~60, the number of micrographs is 10, and the number of independent synthesis batches analyzed is 2. Histograms were generated from measurements of particles selected from multiple TEM images; Figure S8: Size distribution diagram of Sage-derived CDs. The number of particles is ~110, the number of micrographs is 12, and the number of independent synthesis batches analyzed is 2. Histograms were generated from measurements of particles selected from multiple TEM images; Figure S9: FL spectra of Rosemary-derived CDs; Figure S10: FL spectra of Thyme-derived CDs; Figure S11: FL spectra of Sage-derived CDs; Figure S12: UV-VIS spectrum of Olive-derived CDs; Figure S13: UV-VIS spectrum of Rosemary-derived CDs; Figure S14: UV-VIS spectrum of Thyme-derived CDs; Figure S15: UV-VIS spectrum of Sage-derived CDs; Figure S16: XPS spectrum of Rosemary-derived CDs; Figure S17: XPS spectrum of Thyme-derived CDs; Figure S18: XPS spectrum of Sage-derived CDs; Table S1: CHNS elemental analysis.
