# Supplementary material for: Hydrothermal Synthesis of Water-Dispersible Fluorescent Carbon Dots from Renewable Plant Precursors: Antibacterial Activity and Comparative Characterization
Source: Nanomaterials (Basel). 2026 Oct 20;16(20):1328. doi: 10.3390/nano16201328 (PMC13708720; doi:10.3390/nano16201328)
Supplement: Supplementary file 1 [file nanomaterials-16-01328-s001.zip › nanomaterials-4551893-supplementary.pdf]

1. TEM:

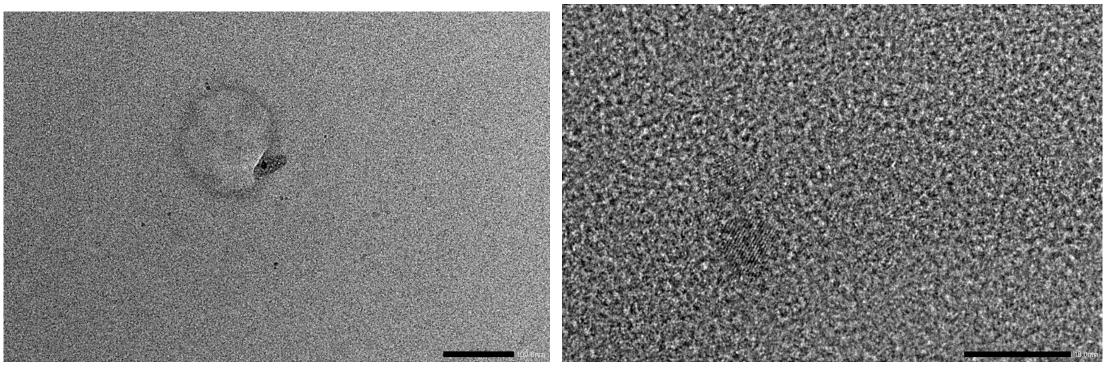

(a) (b)

Figure S1. TEM images of Olive-derived CDs at various magnifications: (a) scale bar = 100 nm, (b) scale bar = 10 nm.

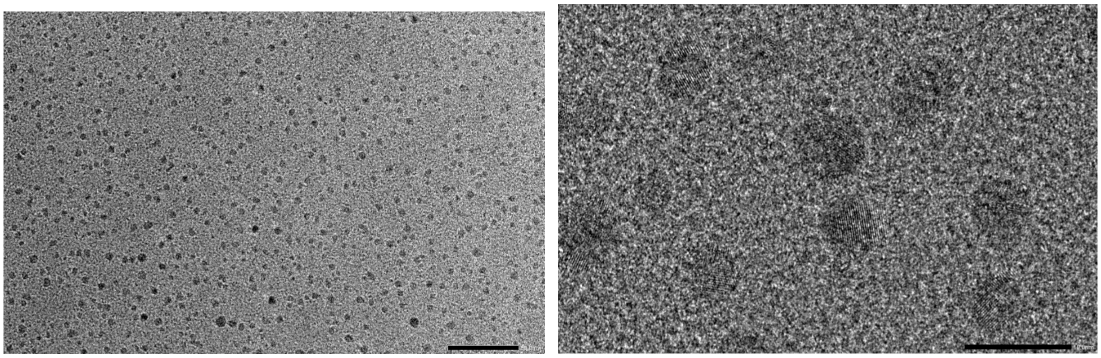

(a) (b)

Figure S2. TEM images of Rosemary-derived CDs at various magnifications: (a) scale bar = 50 nm, (b) scale bar = 10 nm.

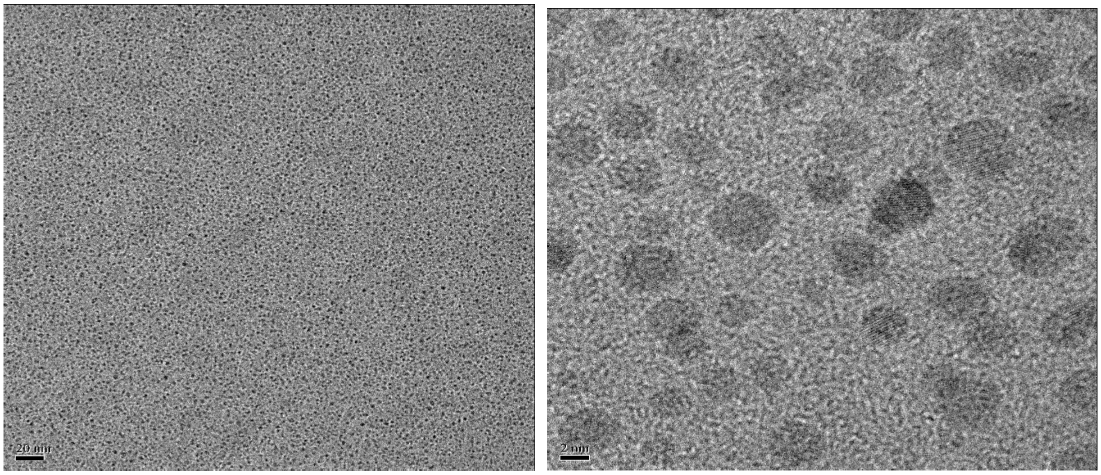

(a) (b)

Figure S3. TEM images of Thyme-derived CDs at various magnifications: (a) scale bar = 20 nm, (b) scale bar = 2 nm.

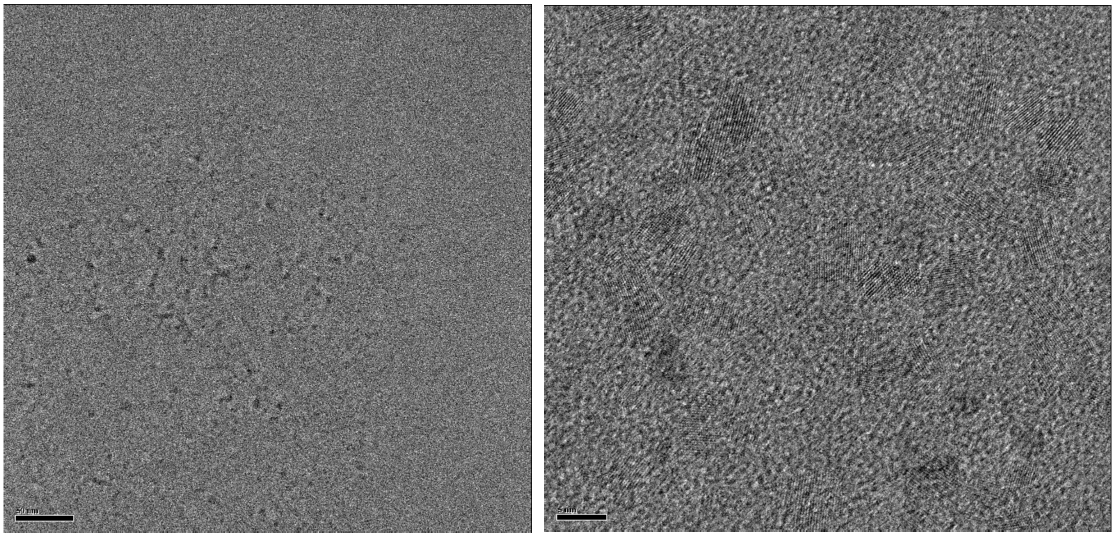

(a) (b)

Figure S4. TEM images of Sage-derived CDs at various magnifications: (a) scale bar = 50 nm, (b) scale bar = 5 nm.

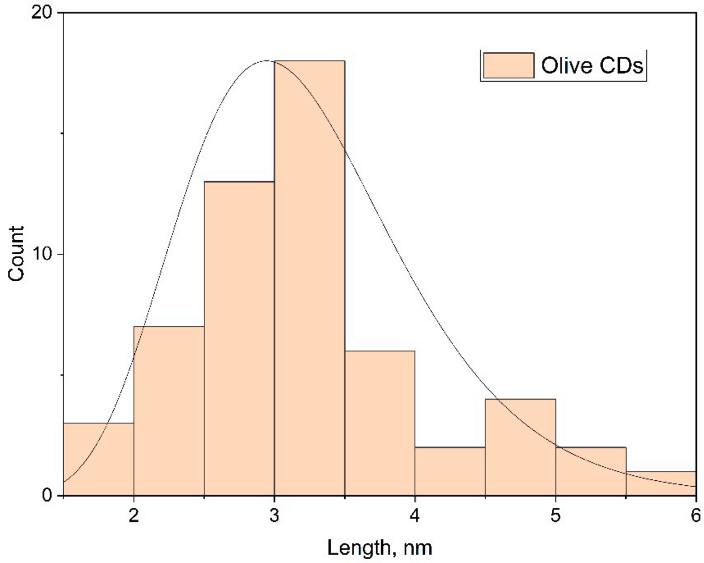

Figure S5. Size distribution diagram of Olive-derived CDs. The number of particles is ~60, the number of micrographs is 15, and the number of independent synthesis batches analyzed is 6. Histograms were generated from measurements of particles selected from multiple TEM images.

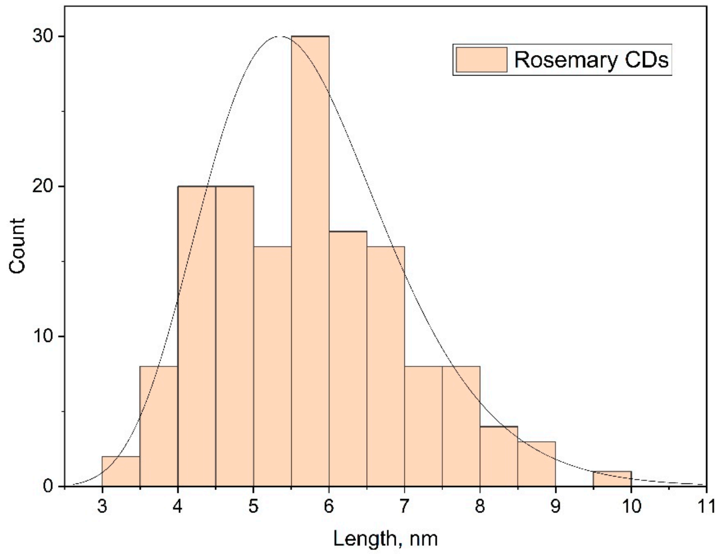

Figure S6. Size distribution diagram of Rosemary-derived CDs. The number of particles is ~90, the number of micrographs is 25, and the number of independent synthesis batches analyzed is 5. Histograms were generated from measurements of particles selected from multiple TEM images.

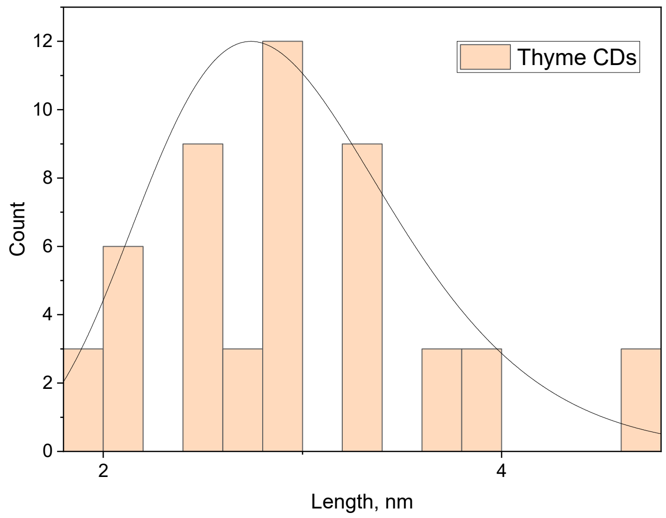

Figure S7. Size distribution diagram of Thyme-derived CDs. The number of particles is ~60, the number of micrographs is 10, and the number of independent synthesis batches analyzed is 2. Histograms were generated from measurements of particles selected from multiple TEM images.

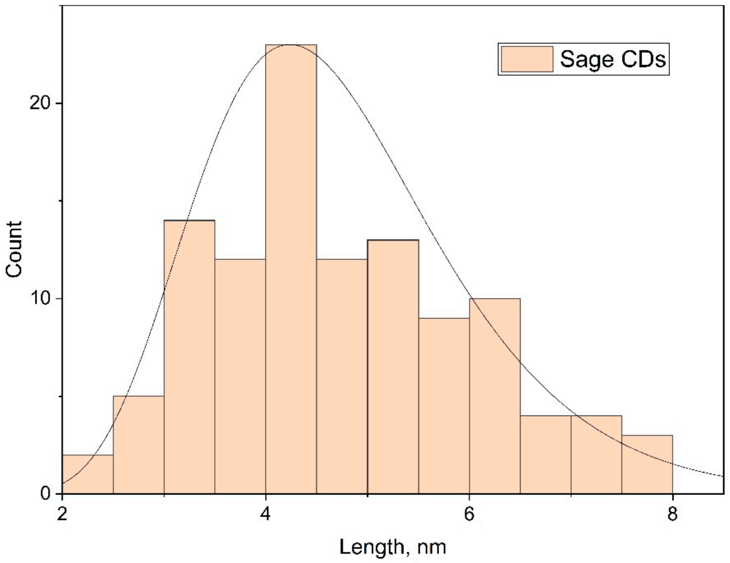

Figure S8. Size distribution diagram of Sage-derived CDs. The number of particles is ~110, the number of micrographs is 12, and the number of independent synthesis batches analyzed is 2. Histograms were generated from measurements of particles selected from multiple TEM images.

2. FL:

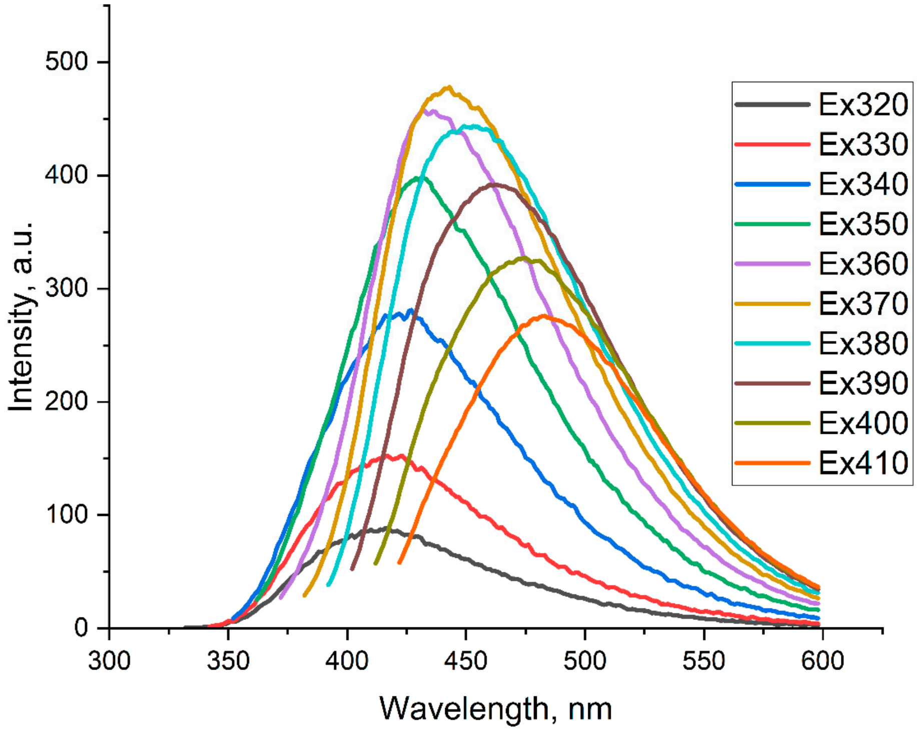

Figure S9. FL spectra of Rosemary-derived CDs.

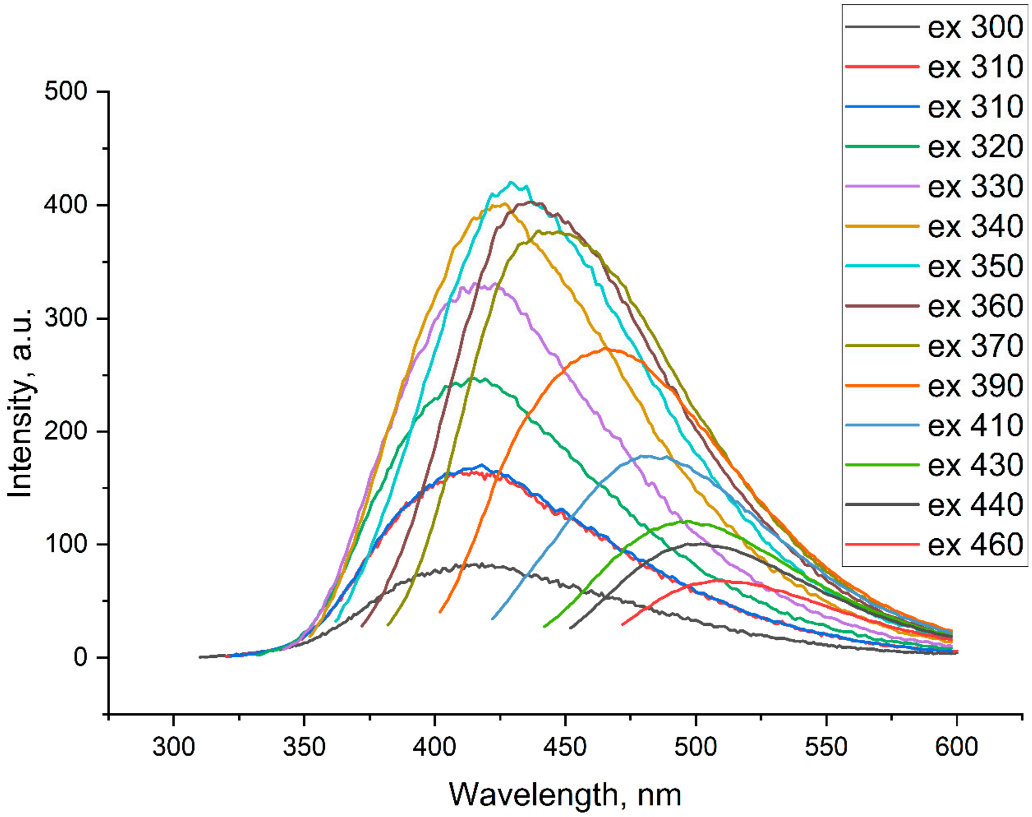

Figure S10. FL spectra of Thyme-derived CDs.

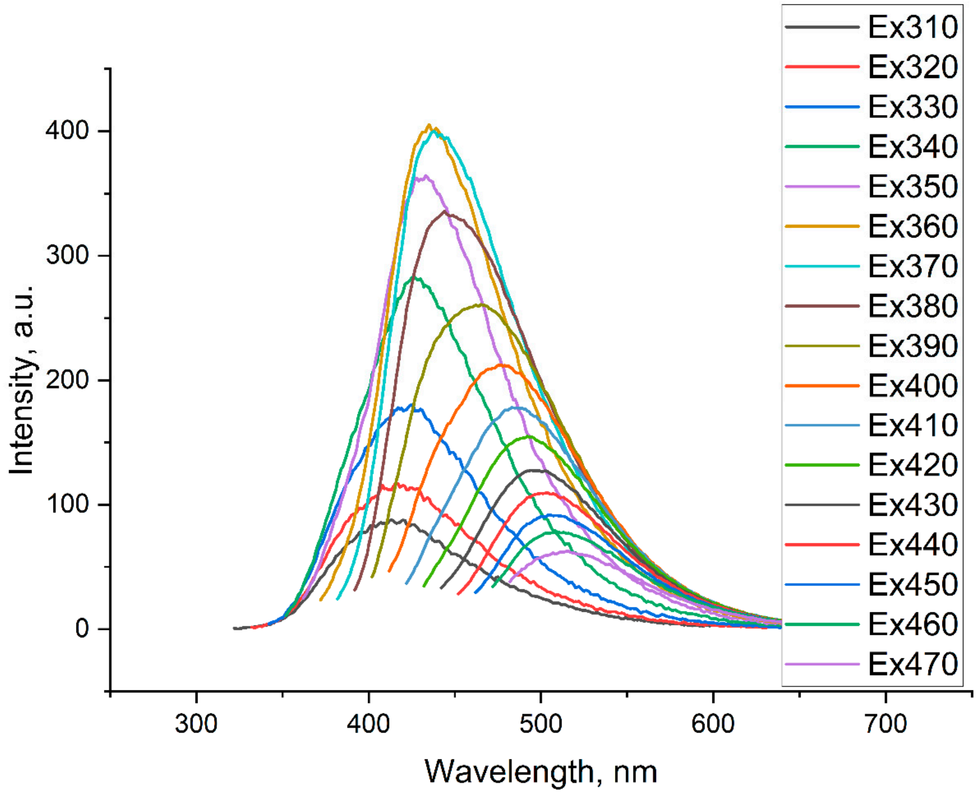

Figure S11. FL spectra of Sage-derived CDs.

Spectra acquisition was performed at a concentration of 0.1 mg/ml. All fluorescence spectra were recorded under identical instrumental settings, including excitation/emission slit widths (10 nm) and detector voltage. No post-acquisition correction was applied to the fluorescence spectra.

3. UV-VIS:

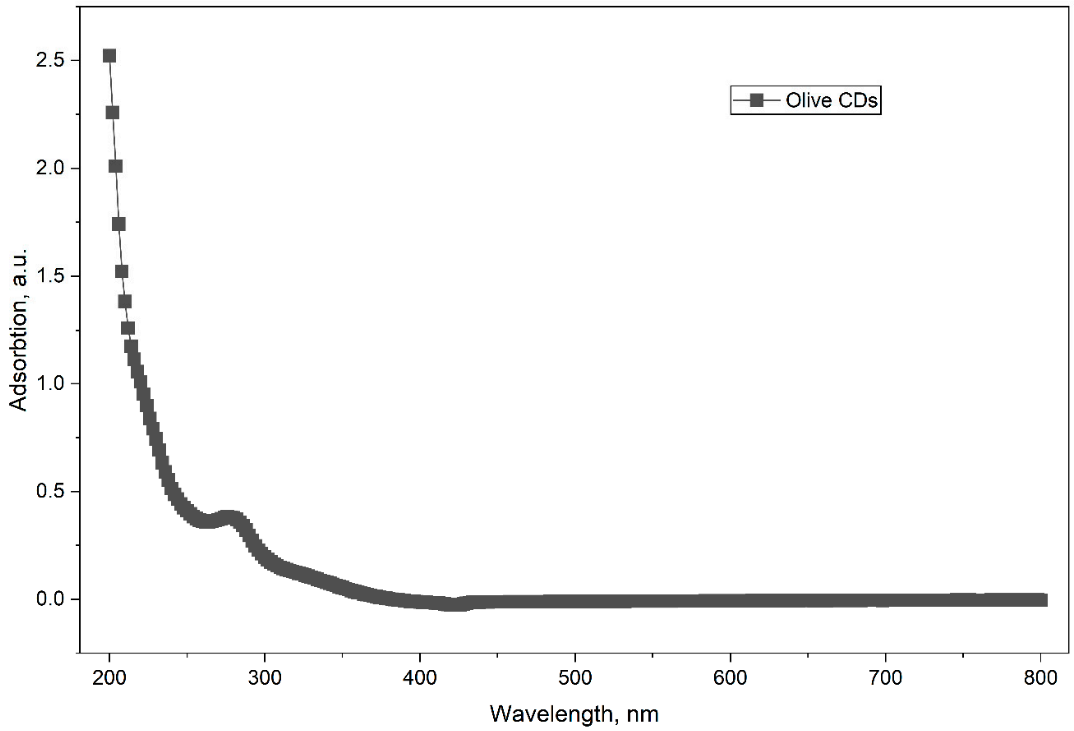

Figure S12. UV-VIS spectrum of Olive-derived CDs.

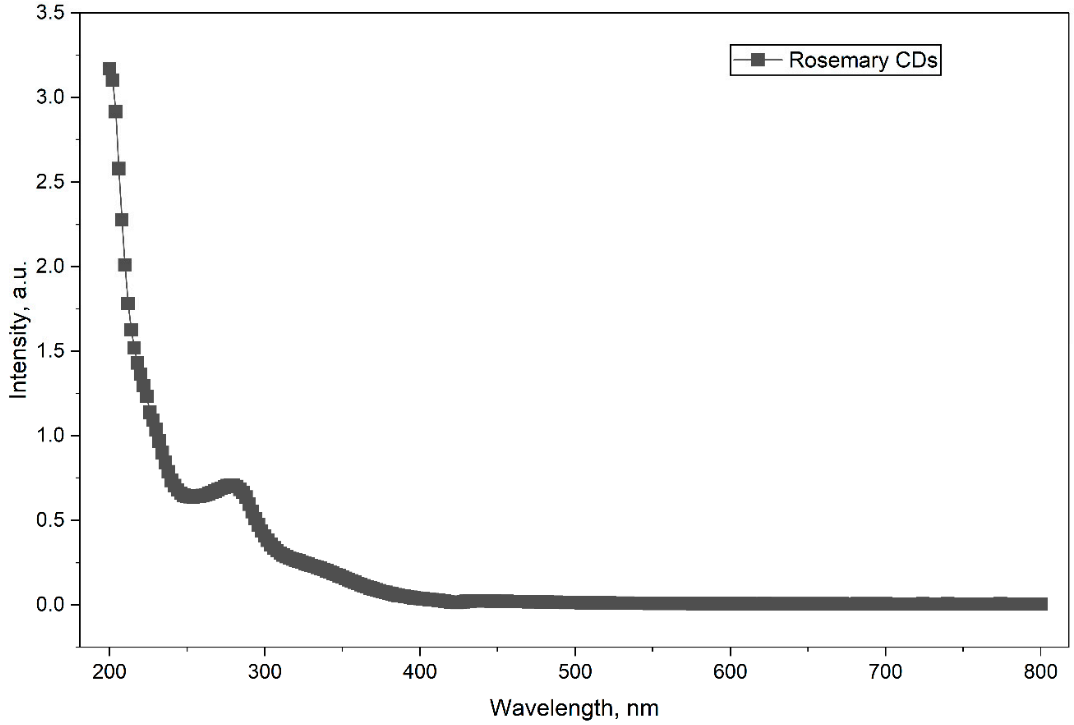

Figure S13. UV-VIS spectrum of Rosemary-derived CDs.

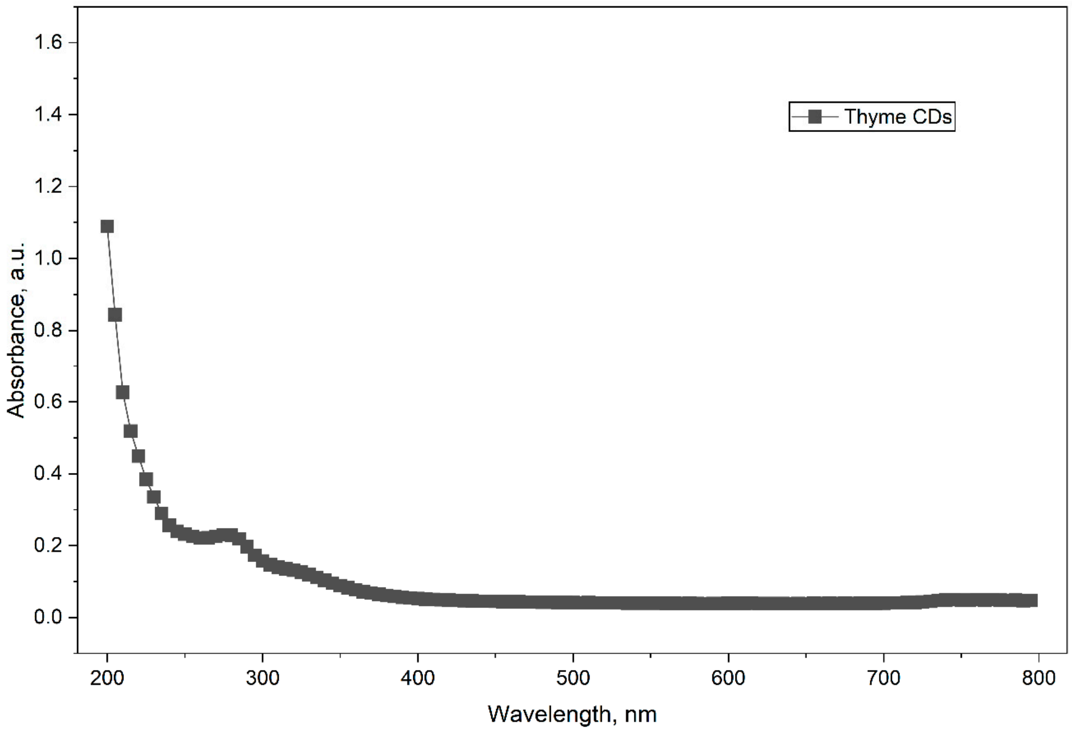

Figure S14. UV-VIS spectrum of Thyme-derived CDs.

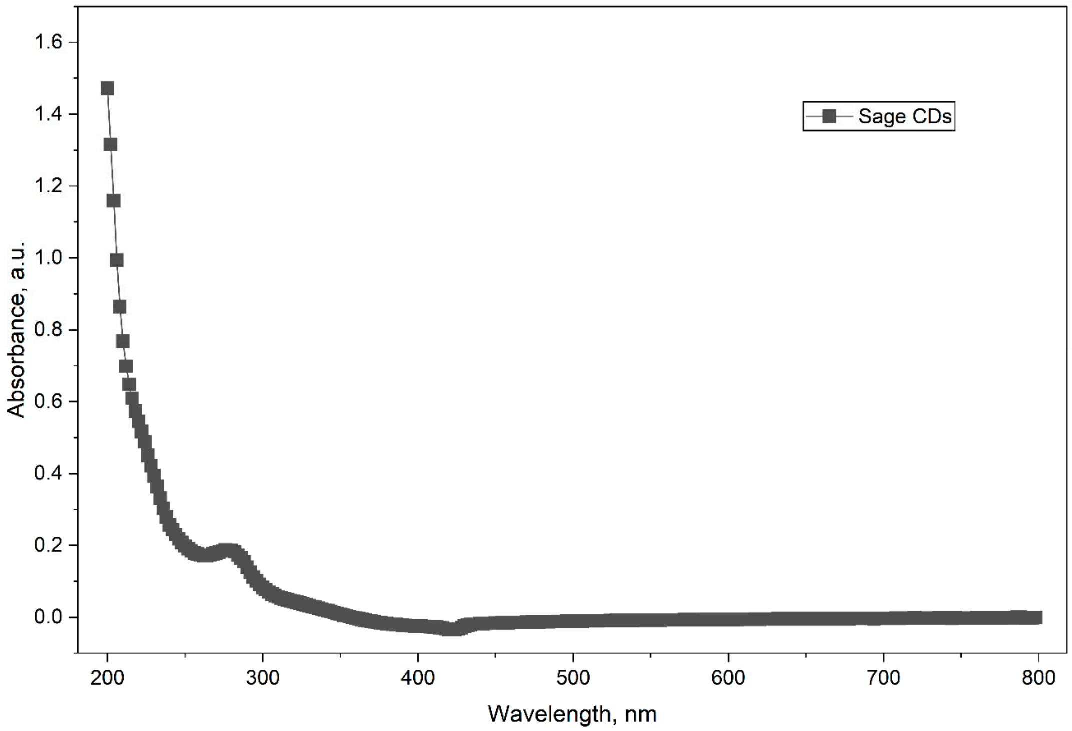

Figure S15. UV-VIS spectrum of Sage-derived CDs.

4. Quantum Yield (QY) measurements:

Quinine sulfate (Q = 0.54 in water) was chosen as the standard fluorescent agent for the determination of QY of synthesized CDs. The quantum yield with reference is calculated by the following equation:

$$Q_{CD} = Q_q \times (I_{CD}/I_q) \times (A_q/A_{CD}) \times (\eta^2_{CD}/\eta^2_q)$$

where Q refers to the quantum yield, I represents the measured integrated emission intensity, A is the optical density, and η is the refractive index of the solvent used. The subscripts 'CD' and 'q' refer to the carbon dots and the reference (quinine sulfate) in this equation. The quantum yield of the carbon dots was measured using an excitation wavelength of 360 nm. To reduce reabsorption effects, the optical densities of the individual solutions in a 1 cm quartz cuvette were kept below 0.1 at the excitation wavelength.

5. XPS:

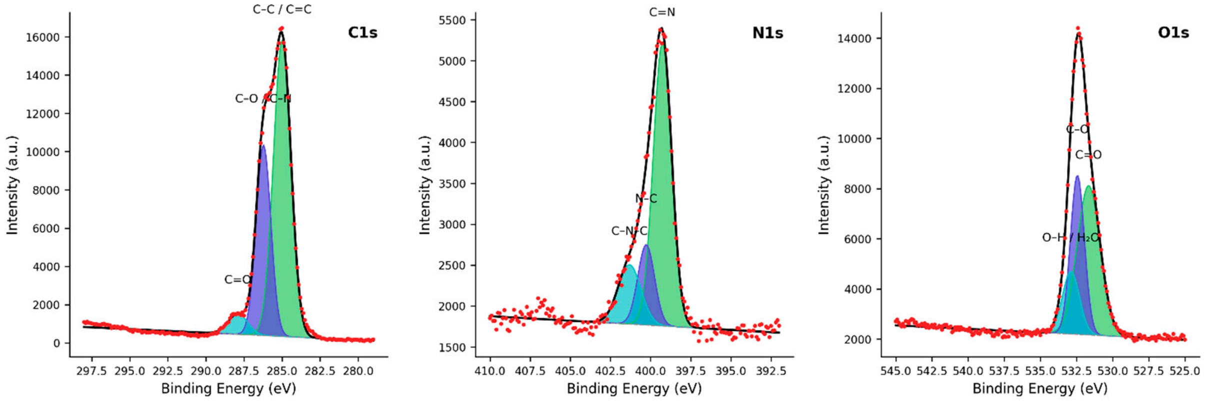

Figure S16. XPS spectrum of Rosemary-derived CDs.

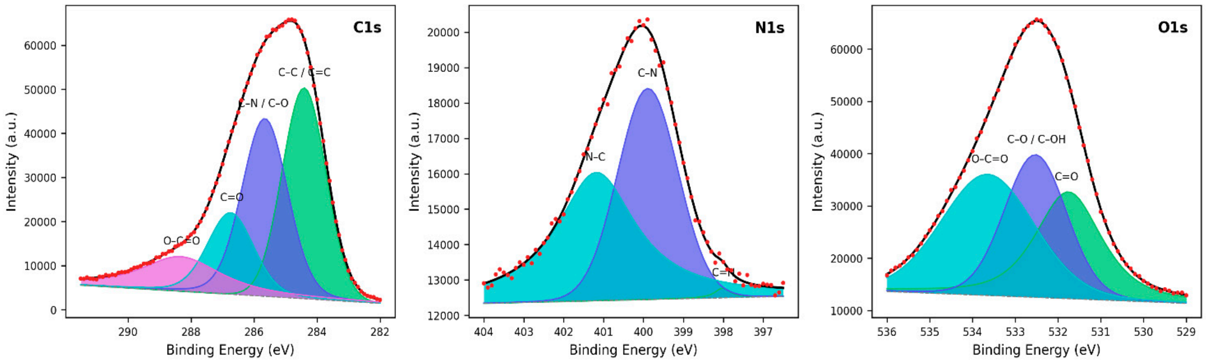

Figure S17. XPS spectrum of Thyme-derived CDs.

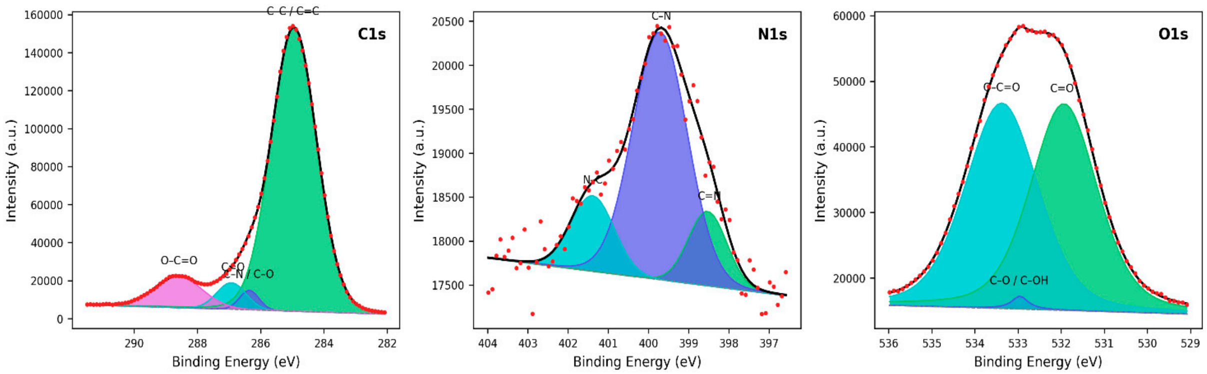

Figure S18. XPS spectrum of Sage-derived CDs.

6. Table S1. CHNS elemental analysis:

CD source	Elemental content, wt.%			
	C	H	N	S
Olive	42.33	7.338	3.812	0.254
Sage	44.28	5.002	5.752	0.112
Rosemary	38.19	5.665	3.125	0.390
Thyme	36.89	5.726	6.182	0.276
